# A Pathogenic Mosaic *TP53* Mutation in Two Germ Layers Detected by Next Generation Sequencing

**DOI:** 10.1371/journal.pone.0096531

**Published:** 2014-05-08

**Authors:** Sam Behjati, Mariana Maschietto, Richard D. Williams, Lucy Side, Mike Hubank, Rebecca West, Katie Pearson, Neil Sebire, Patrick Tarpey, Andrew Futreal, Tony Brooks, Michael R. Stratton, John Anderson

**Affiliations:** 1 Cancer Genome Project, Wellcome Trust Sanger Institute, Wellcome Trust Genome Campus, Hinxton, Cambridgeshire, United Kingdom; 2 Department of Paediatrics, University of Cambridge, Cambridge, United Kingdom; 3 Unit of Molecular Haematology and Cancer Biology, UCL Institute of Child Health, London, United Kingdom; 4 Departments of Clinical Genetics, Great Ormond Street Hospital, London, United Kingdom; 5 Departments of Histopathology, Great Ormond Street Hospital, London, United Kingdom; Cancer Research Centre of Lyon, France

## Abstract

**Background:**

Li-Fraumeni syndrome is caused by germline *TP53* mutations and is clinically characterized by a predisposition to a range of cancers, most commonly sarcoma, brain tumours and leukemia. Pathogenic mosaic *TP53* mutations have only rarely been described.

**Methods and Findings:**

We describe a 2 years old child presenting with three separate cancers over a 6 month period; two soft tissue mesenchymal tumors and an aggressive metastatic neuroblastoma. As conventional testing of blood DNA by Sanger sequencing for mutations in *TP53, ALK, and SDH* was negative, whole exome sequencing of the blood DNA of the patient and both parents was performed to screen more widely for cancer predisposing mutations. In the patient's but not the parents' DNA we found a c.743 G>A, p.Arg248Gln (CCDS11118.1) *TP53* mutation in 3–20% of sequencing reads, a level that would not generally be detectable by Sanger sequencing. Homozygosity for this mutation was detected in all tumor samples analyzed, and germline mosaicism was demonstrated by analysis of the child's newborn blood spot DNA. The occurrence of separate tumors derived from different germ layers suggests that this *de novo* mutation occurred early in embryogenesis, prior to gastrulation.

**Conclusion:**

The case demonstrates pathogenic mosaicim, detected by next generation deep sequencing, that arose in the early stages of embryogenesis.

## Introduction

Germline mutations in the *TP53* gene cause Li-Fraumeni syndrome (LFS, OMIM 151623), an autosomal dominant highly penetrant cancer predisposition syndrome characterized by a variety of early onset tumors [1], [2], [3]. LFS is associated with an increase in overall cancer incidence in affected individuals and an early age of cancer onset [4]. Many patients are affected in early childhood, most often by sarcomas, brain and adrenocortical tumors [5]. However, there are many families with a hereditary predisposition to cancer suggestive of LFS who do not meet the classical clinical diagnostic criteria and yet still carry *TP53* mutations; less rigorous criteria were therefore developed [6], [7], [8].

Different mutant p53 proteins may have diverse functional and biological effects, which could partially explain the heterogeneity reported between Li-Fraumeni families [9]. Tumor spectrum, severity and phenotype can be associated with other germline genetic factors [10], [11] and/or increase in DNA copy number variation [12], [13].

While 70% of the patients diagnosed with classical LFS have mutations in *TP53* [3], less than 20% of cases classified using less stringent criteria carry mutation in the gene. This raises the possibility of other causative germline gene mutations for LFS, or of patients with mosaicism for germline mutations, undetectable using current methods for *TP53* screening. Identification of *TP53* mutation has important clinical implications for early detection and treatment of associated neoplasms through clinical surveillance [14].

Improved sequencing technologies offer unprecedented opportunities for investigating the role of rare genetic variation in common disease. The current study provides new insights into the role of mosaic genetic variants in cancer, and the use of sequencing technologies for their identification. We describe a child presenting with metastatic neuroblastoma and two soft tissue tumors within the first 2 years of life, in the absence of a family history of cancer. Germline mutation of *TP53* was undetectable by conventional Sanger sequencing but identified by whole exome sequencing. This mutation was screened in three tumors, which displayed high rates of the mutated allele. Further analysis in the blood from the child's neonatal blood spot test (Guthrie card) showed the mutation was present at birth with the same mosaic pattern.

## Materials and Methods

### Patient samples

For using of parents and child samples, written informed consent for germline genetic analysis, including whole exome sequencing, was obtained from the parents of the index case patient in this study using standard UK clinical National Health Service consent procedures. Research Governance approval for detailed genetic analysis was provided by the Great Ormond Street Hospital Research and Development Department (approval 11MH09).

### DNA and RNA extraction

DNA from frozen samples and Guthrie card was extracted with QIAamp DNA Mini Kit (Qiagen) following the manufacturer's protocol. Paraffin blocks were cut as 8 µm sections on plain glass slides. Targeted regions for sampling were marked on adjacent hematoxylin and eosin sections by the study pathologist and recovered by scrape macrodissection. Between 3 and 20 sections were macrodissected depending on the tissue sample's size. DNA from formalin-fixed paraffin embedded sections was extracted with QIAamp DNA FFPE Tissue Kit (Qiagen) according to the manufacturer's instructions. RNA from frozen tumour sample was extracted using the RNeasy minikit (Qiagen) and cDNA synthesized using the SuperScript III (Invitrogen), using standard protocols.

### Whole exome sequencing

Whole exome sequencing from genomic (native) DNA of the child (sample PD9058b) and his parents was performed by BGI, Shenzhen, China, on an Illumina HiSeq DNA Analyzer using Agilent SureSelect Human All Exon 50 Mb for target enrichment. Forty to fifty percent of the target region had a minimum coverage of 30X. Sequencing reads were aligned to the human genome (NCBI build 37) using the BWA algorithm on default settings. Reads which were unmapped, PCR-derived duplicates or reads outside the targeted region of the genome were excluded from the analysis.

### Variant calling

The CaVEMan (cancer variants through expectation maximization) algorithm was used to call single nucleotide substitutions [15]. To call insertions and deletions, we used split-read mapping implemented as a modification of the Pindel algorithm [15]. Mutations were annotated to Ensembl version 58. Post-processing filters were applied to the output to improve specificity. Variants of the final data set were inspected manually to identify potentially genuine variants. A single candidate variant was identified, which was validated as described below.

### Validation by targeted resequencing

Two independent samples of genomic (native) DNA derived from peripheral blood of the child (PD9058b3, PD9058b4) as well as parental DNA were enriched for *TP53* through a custom-made bait set (Agilent) and sequenced on an Illumina MiSeq Personal Sequencer.

### Sanger sequencing and Deep PCR amplicon sequencing

Primers flanking *TP53* exon 7, where the mutation is located, were retrieved from the IARC TP53 database. PCR reactions were performed using the Platinum Taq DNA Polymerase PCR kit (Life Technologies). Amplicons were sequenced using the BigDye terminator cycle sequencing kit and an ABI 3130 automated sequencer (Applied Biosystems). Sequencing traces were analyzed with GeneScreen (http://dna.leeds.ac.uk/genescreen/) followed by visual inspection, with reference to the human genome reference sequence, build hg19/GRCh37 (http://genome.ucsc.edu). PCR-amplification and Sanger sequencing of the cDNA were carried out with primers in flanking exons 6 and 8. All primers were tagged for library construction (sequences available on request).

Indexed libraries of the PCR products were prepared using the Illumina Nextera XT library preparation kit and protocol, and were sequenced with the Illumina MiSeq system, generating 2×150-bp reads. Only sequences that passed quality control coverage metrics were used, generating median coverage greater than 200,000 for DNA samples and 100 for the cDNA sample across *TP53* exon 7 (average median coverage 113,222x). The ultra-deep sequence data were aligned to the reference genome and variants called using the standard MiSeq Reporter pipeline. Variant quality and sequence read counts were then assessed using the Integrative Genomics Viewer (IGV) version 2.3 [16].

### Immunohistochemistry

One tumor-rich section per case was selected for immunohistochemical analysis of *TP53.* 4 µm sections of FFPE tissue were stained with mouse anti-human monoclonal antibody (clone DO-7 from Leica, cat# PA0057) using the Leica BOND-MAX automated system. Antigen retrieval was performed using the BOND-MAX heat induced epitope retrieval 2 program for 20 minutes. Staining was evaluated by the pathologist.

## Results

### Clinical presentation with multiple tumours

The patient, a thriving male infant, presented at the age of 9 months with a lump in his left groin, which was excised and diagnosed as a benign myofibroblastic proliferation. He had no relevant past medical history, and there was no family history indicative of an underlying cancer predisposition syndrome. Two months later he re-presented with a lump in the ipsilateral pelvic/inguinal region. The histological appearance this time was of sarcoma NOS, with tumor close to the resection margins but no evidence of metastatic disease on staging investigations. He received adjuvant chemotherapy (Vincristine and Dactinomycin) for 5 months. End of treatment imaging revealed a mass adjacent to the right kidney, a biopsy of which showed neuroblastoma with *MYCN* amplification (hundreds of copies of *MYCN* probe in double minute pattern). A staging MIBG scan identified widespread metastatic disease. Treatment for high risk neuroblastoma was therefore commenced with first and second line induction chemotherapy. He then developed an orbital swelling caused by an underlying mass, which was shown to be a neuroblastoma on biopsy. *MYCN* amplification this time, however, was seen in homogeneously staining regions rather than in the double minute pattern, which raised the possibility of metachronous neuroblastoma, rather than metastatic progression. The child failed to respond to further treatment, and died at age 26 months.

### Evaluation of germline and tumour *TP53* mutation status

**Figure 1:**
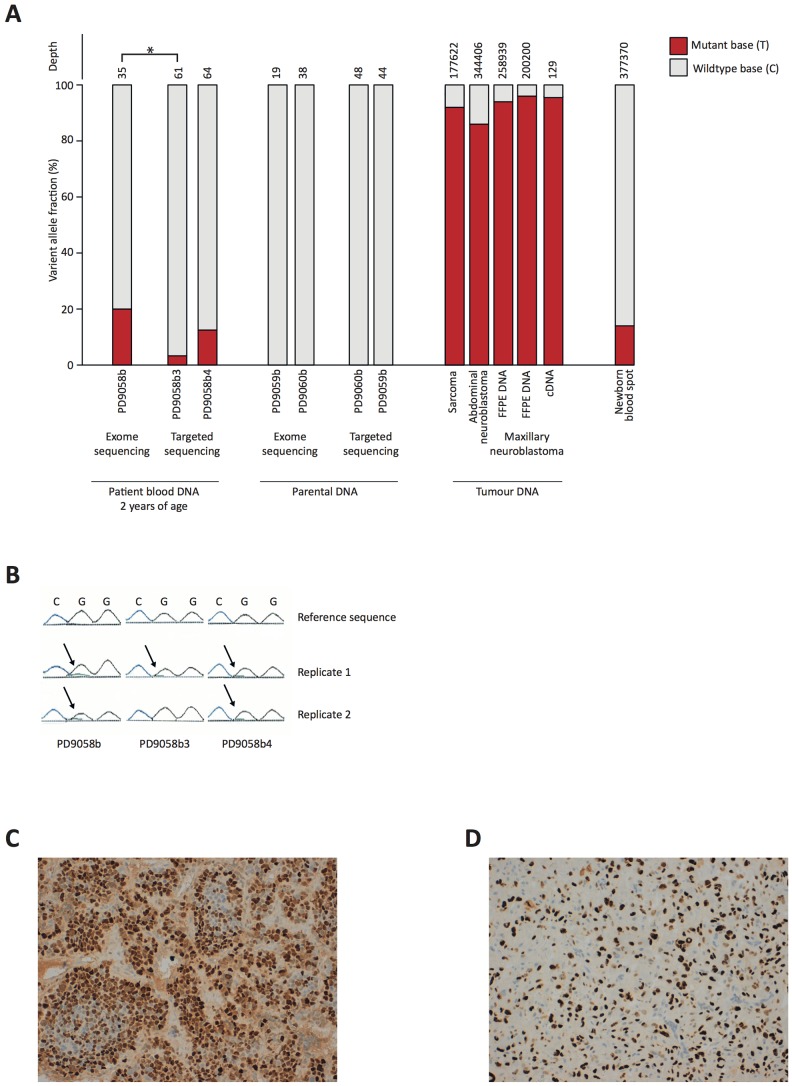
**A.** Mutant read frequency in different samples. The patient's blood samples were all taken around the time of neuroblastoma diagnosis. Numbers of sequencing reads per sample are displayed in the top level. * p = 0.01, comparing mutant read count frequencies between samples PD9058b3 and PD9058b with Fisher's exact test. Differences between PD9058b2 and sample b/b3 are not significant (p>0.05). **B.** Capillary sequencing of control germline DNA (Reference sequence) and patient's blood samples extracted at three time points, showing very low level of adenine (black arrow). The guanine (black peak) dropped approximately between 8–17% relative to control, as given by the software, which would not usually be classed as significant when looking for heterozygous germline mutations. **C and D.** Immunohistochemistry to show strong nuclear staining p53 in neuroblastoma (C) and sarcoma NOS (D).

As part of a screen for known cancer predisposition syndromes, DNA isolated from both the child's and his parents' blood was sequenced by capillary electrophoresis in a clinical diagnostic laboratory to detect mutations in known cancer associated genes, including *TP53, ALK* and *SDH*. No mutations were found by conventional analysis of sequencing data in the child or his parents. Whole exome sequencing of the child's and his parent's DNA samples was performed to screen more widely for pathogenic germline mutations. Sequence analysis revealed a c.743 G>A, p.Arg248Gln (CCDS11118.1) variant in *TP53*, a pathogenic *TP53* mutation known to cause Li Fraumeni syndrome. The mutation was absent from the parents' DNA. Interestingly, the variant was present in only approximately 20% of sequencing reads, suggesting that the patient was mosaic for *TP53* (**Figure 1A**). To validate the variant, DNA samples from three separate blood samples of the child were re-sequenced by conventional capillary sequencing in the diagnostic laboratory and the data this time manually inspected. A low level variance of the variant base was seen in all three samples. These levels, if detected, would not normally be reported as significant (**Figure 1B**).

Deep sequencing of the coding sequence of *TP53* was performed in two DNA samples of the child's blood as well as each of the parents' blood DNA, using custom-made bait capture for enrichment of target regions, which confirmed the presence of the variant as mosaic in the child (variant allele fraction 3.3% and 12.5%), and its absence from parental DNA (**Figure 1A**).

Ultra deep sequencing for the target region by PCR was next performed on DNA derived from blood of the patient's newborn blood spot (Guthrie) Card, from the two frozen neuroblastoma tumor samples, from the formalin-fixed paraffin embedded sarcoma NOS sample, and from one matched cDNA sample from the sarcoma. Analysis of the Guthrie card showed that around 14% of the blood cells had the mutant allele at the time the child was born. In the tumor cases the results showed homozygosity (range 86 to 97%) for the variant, consistent with genomic loss of the wild type allele as a tumour promoting event in common between the different cancers (**Figure 1B**). Immunohistochemistry of p53 disclosed strong nuclear staining in all tumors, typical of *TP53* mutations that interfere with p53 protein function (**Figure 1C,D**).

## Discussion

We present the case of a young boy with severe Li Fraumeni syndrome who has a *de novo* constitutional *TP53* variant that is mosaic and affects at least two germ layers. Co-occurrence of a normal cell line and a cell line carrying the mutation constitute a mosaicism, which can be associated only with post-zygotic *de novo* mutations [17]. To our knowledge, we have demonstrated the earliest documented occurrence of a cancer initiating *TP53* point mutation in human embryogenesis. Furthermore, our report illustrates the utility of, and necessity for, taking next generation sequencing (NGS) technologies into clinical practice.

Studies of human *in vitro* fertilized embryos show that mosaicism is common from development to adulthood [18]. The use of more sensitive methods is increasing detection of genome variation in both disease and in healthy individuals, as demonstrated by copy number alterations, genomic rearrangements single nucleotide variation, repeat expansions and microsatellite instabilities [18], [19], [20], [21].

Of biological interest is that the variant drives the formation of cancers derived from two different germ layers. Therefore, a developmental stage during which the variant arose can be defined. Neuroblastoma arises from the neural crest, which is derived from ectoderm. Sarcomas and blood cells are of mesodermal origin. The *TP53* variant therefore must have arisen in an embryonic cell that contributed to both the mesodermal and the ectodermal lineages, which segregate during gastrulation. This implies that the mutation arose prior to formation of the three germ layers.

A reappraisal of the incidence of mosaicism has been made possible by new sequencing technologies [18]. It is interesting in this context that human embryos analyzed by single cell genomic analysis have recently been shown to have a high incidence of abnormal chromosome complements [22]. Previously, a case of constitutional *TP53* mosaicism also with a severe phenotype was observed in a girl who developed two malignancies of mesodermal origin, between the ages of one and five years [17]. This patient's variant (R282W) was detectable by capillary sequencing and shown to be absent from ectodermal tissue. Other cancer-predisposing monogenetic diseases in which pathogenic mosacisim has been documented include neurofibromatosis and familial adenomatous polyposis [23], [24], [25], [26] though mosacisim was associated with less severe phenotypes in these diseases. *RB1* mosaicism, on the other hand, has also been reported to cause a high expressivity phenotype [27].

The practical aspect this report highlights is the limitation of the use of Sanger sequencing in clinical practice to detect pathogenic germline variants. Furthermore, using NGS techniques, quality control frequency filters tuned to detect heterozygous variants may be too stringent to identify mosaic variants. Indeed standard processing filters of two independent variant calling algorithms excluded the *TP53* mutation on initial analysis.

Of note, by sequencing four different DNA samples of the child, we were able to demonstrate variation of the variant allele fraction, ranging from 3.3 to 20%, with significant differences observed between two samples. This indicates that the relative number of circulating blood cell clone may be changeable, thus presenting further challenges for routine detection. However, reliable identification of germline *TP53* mutation even at a low mosaic level is very important because it affects clinical cancer management, with many clinicians opting to avoid radiotherapy or DNA damaging agents if possible. We speculate that as deep sequencing replaces Sanger sequencing in clinical genetics laboratories more cases of genetic diseases driven by mosaic mutations will surface.
